# A Fast and Implantation-Free Sample Production Method for Large Scale Electron-Transparent Metallic Samples Destined for MEMS-Based In Situ S/TEM Experiments

**DOI:** 10.3390/ma14051085

**Published:** 2021-02-26

**Authors:** Matheus A. Tunes, Cameron R. Quick, Lukas Stemper, Diego S. R. Coradini, Jakob Grasserbauer, Phillip Dumitraschkewitz, Thomas M. Kremmer, Stefan Pogatscher

**Affiliations:** Chair of Non-Ferrous Metallurgy, Montanuniversitaet Leoben, 8700 Leoben, Austria; matheus.tunes@gmail.com (M.A.T.); lukas.stemper@unileoben.ac.at (L.S.); diego.s.r.coradini@unileoben.ac.at (D.S.R.C.); jakob.grasserbauer@unileoben.ac.at (J.G.); phillip.dumitraschkewitz@unileoben.ac.at (P.D.); thomas.kremmer@unileoben.ac.at (T.M.K.); stefan.pogatscher@unileoben.ac.at (S.P.)

**Keywords:** MEMS, protochips fusion, in situ S/TEM, sample preparation method

## Abstract

Microelectromechanical systems (MEMS) are currently supporting ground-breaking basic research in materials science and metallurgy as they allow in situ experiments on materials at the nanoscale within electron microscopes in a wide variety of different conditions such as extreme materials dynamics under ultrafast heating and quenching rates as well as in complex electro-chemical environments. Electron-transparent sample preparation for MEMS e-chips remains a challenge for this technology as the existing methodologies can introduce contaminants, thus disrupting the experiments and the analysis of results. Herein we introduce a methodology for simple and fast electron-transparent sample preparation for MEMS e-chips without significant contamination. The quality of the samples as well as their performance during a MEMS e-chip experiment in situ within an electron microscope are evaluated during a heat treatment of a crossover AlMgZn(Cu) alloy.

## 1. Introduction

The use of microelectromechanical systems (MEMS) within scanning/transmission electron microscopes (S/TEM) is at the forefront of experimental science, particularly in the fields of nanotechnology and materials science. As a rapidly evolving and emerging technology, MEMS experiments with in situ TEM can provide countless opportunities for investigation of the real-time response of materials in corrosive and gaseous environments [1,2], under extreme dynamic changes when subjected to ultrafast heating and cooling rates of up to 106 K·s^−1^ [3,4], under mechanical loading [5,6] or when subjected to complex photocatalytic environments [7,8]. These experiments are now in fact contributing to the design of new materials at the nanoscale as well as supporting the progress of basic research in science by allowing complex physicochemical [9] and/or elastoplastic [10] mechanisms to be fundamentally investigated at the nanoscale.

A major challenge when carrying out MEMS experiments with in situ TEM lies in the sample preparation methodology chosen for producing good-quality, electron-transparent lamellae and their subsequent transfer to the MEMS e-chips. Up to now, such sample preparation methodology has been highly dependent on the application of dual-beam scanning electron microscopes (SEM) with focused ion beam (FIB) capabilities [4,11,12].

Unquestionable reliability and efficiency are evident characteristics of FIB-based methods for producing electron-transparent samples from metallic substrates [13], but several degradation mechanisms are reported to occur during the stages’ sample preparation within SEM-FIBs [14]. These may impact the final quality of a specimen and possibly affect the reliability of the results generated during MEMS experiments within a S/TEM. Many metallurgical samples, such as those made from Al-based alloys, may strongly interact with either Ga ions [15] or Pt/C layers often used as a top-protective coatings [14,16], resulting in contamination and subsequent formation of undesirable artefacts. The advent of plasma-based FIBs (using Xe ions) is reported to mitigate some deleterious effects found in Ga-based FIBs [15], but the use of Xe ions can impact electron-transparent metallic lamellae in different ways including radiation-induced damage or even the formation of nanometer-sized Xe bubbles [17].

Given the facts, the optimal scenario would be an electron-transparent sample preparation methodology free of both implantation from ballistic cascades (Ga, Xe) and contamination from welding (Pt). We report in this paper an alternative methodology for producing good-quality and implantation-free electron-transparent metallic specimens for MEMS experiments with in situ S/TEM, consisting, in essence, of a series of scalpel cuts on an electropolished 3 mm disk to isolate a suitably sized sample. Following a detailed description of the MEMS electron-transparent sample preparation methodology, a heat treatment experiment in situ within a S/TEM is presented using a MEMS chip and the quality of the produced specimen before and after the experiment is evaluated using conventional and analytical electron-microscopy techniques.

## 2. Materials and Methods

### 2.1. Provenance of the Metallic Samples

The MEMS sample preparation methodology reported in this research works for a wide variety of metallic samples. As a demonstrative example, the experiments reported in this present paper use a novel crossover AlMg_4.7_Zn_3.6_Cu_0.6_ alloy (in wt.%). Throughout the text, these samples will be referred to as “AlMgZn(Cu) alloy”. The alloy was pre-aged for 3 h at 373 K and subjected to a minor deformation level of 2% (for details on the synthesis, processing and materials properties we refer to [18]).

### 2.2. Jet Electropolishing (JEP)

Electron-transparent specimens of the AlMgZn(Cu) alloy were prepared using the technique of jet electropolishing. The samples were polished and ground to 100 µm of thickness and mechanically punched out to 3 mm disks. For the JEP procedure, an electrolyte solution composed of 25% nitric acid and 75% methanol (in vol.%) was used at a temperature range of 253–257 K with the electrode potential set to 12 V. During JEP, the specimen current slightly oscillated around 90 mA. After JEP, the samples were washed in three different pure methanol baths and left to dry in air.

### 2.3. Scanning/Transmission Electron Microscopy (S/TEM)

Electron microscopy was carried out using a Thermo Fisher Scientific™ Talos F200X scanning/transmission electron microscope (ThermoFisher Scientific, Hillsboro, OR, USA). The microscope operates a X-FEG filament (a refinement of the Schottky thermally assisted field emission gun) at 200 kV and features the Super-X energy dispersive X-ray (EDX) spectroscopy technology. For the investigations reported in this paper, the following imaging modes were used: bright-field TEM (BF-TEM), selected-area electron diffraction (SAED), low-angle annular dark-field (LAADF) and bright-field STEM (BF-STEM).

### 2.4. MEMS Experiments with In Situ S/TEM

In situ S/TEM heat treatment experiments were performed using a Protochips FUSION 200 MEMS chip-based holder (Protochips, Morrisville, NC, USA). with double-tilt capability. For the heat treatment experiments reported in this work, the AlMgZn(Cu) alloy was subjected to a heating ramp of +60 K·min^−1^ up to 458 K where the samples were held for 1200 s. Then, a cooling ramp of −60 K·min^−1^ was applied down to a temperature of 298 K. This heat treatment specification is denoted in the metallurgical literature as a paint bake [18]. For the MEMS experiments reported in this work, e-chips without coating on the SiN membrane (a hollow region with 9 holes where the electron-transparent piece was placed to be analysed within the S/TEM) were used.

## 3. Results and Discussion

### 3.1. MEMS Sample Preparation Methodology

**Figure 1:**
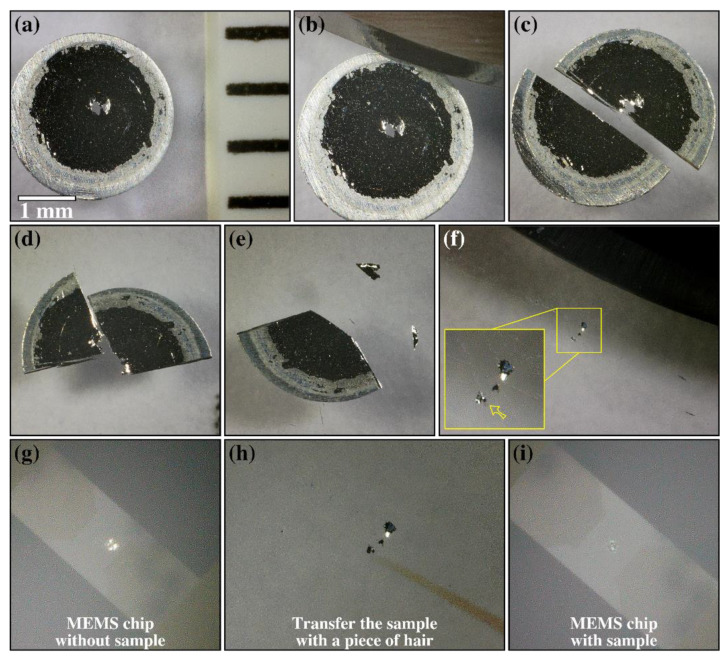
Step-by-step description of the sample preparation methodology proposed in this work. The optical micrograph in (**a**) shows the 3 mm disk of the electropolished AlMgZn(Cu) alloy with a central hole where the electron-transparent regions are located. Image (**b**) includes the curved scalpel used to make the cuts in the field of view. Steps(**c**–**e**) show the subsequent cutting made with a sharp laboratory scalpel: note the cuts are made to separate the regions-of-interest around the central hole from the whole 3 mm disk. The optical micrograph in (**f**) shows three pieces of an electron-transparent area that have been cut at higher magnification: a mid-sized piece of size around 50 µm as indicated by the yellow arrow in the inset in (**f**) was selected for transfer onto the microelectromechanical systems (MEMS) chip. The optical micrograph in (**g**) shows the MEMS chip without the sample in its membrane (with holes). The sample is then transferred (**h**) to the MEMS chip by using a piece of brush-bristle with intrinsic static after frictional static charging. The sample is positioned on the MEMS chip membrane as shown in (**i**). The leftover pieces (from the inset in (**f**)) can still be used to produce additional samples in different MEMS e-chips.

The MEMS sample preparation methodology investigated in this work is described in the set of optical micrographs in Figure 1a–i. The entire process can be performed using a simple stereo microscope with magnification in the order of 100–200×.

The methodology consists of selecting an electropolished 3 mm disk that was pre-confirmed to have electron-transparent areas around its central hole as shown in Figure 1a. These transparent areas are the regions-of-interest (ROI) for the MEMS experiments with in situ S/TEM. The 3 mm disk is placed onto a glass slide. Then, with a sharp laboratory scalpel, a series of cuts are performed on the 3 mm disk in order to isolate parts of the ROI as shown in the set of optical micrographs in Figure 1b–e. The samples prepared in this work used a polished sapphire slide as a cutting surface. Sapphire’s high hardness means that no visible scalpel scratches result from the cutting process, which, in addition to visible and tactile benefits, means much fewer potential contaminate particles originate from the cutting surface. The general experience with conventional laboratory glass slides is decent, however, especially with softer materials, and is an adequate choice for this procedure. For optimal results when making the cuts, the authors advise using a curved scalpel and aligning the intended cut while the scalpel tip is in contact with the cutting surface. Firmly lowering the scalpel handle from this position provides the best control and allows the precision necessary to isolate the ROI. With the ROI cut into smaller pieces of around 50–100 µm as denoted, one piece is selected to be transferred onto the MEMS chip as shown in the inset of the optical micrograph in Figure 1f.

With the electron-transparent piece (≈50 µm) cut and selected from the ROI, the MEMS chip is placed into the field-of-view: the membrane of the MEMS chip can be seen in the stereo microscope as shown in Figure 1g, and this is the target area for the electron-transparent piece. The transfer procedure is performed with the use of an animal hair (taken from a regular high-quality paint brush commonly found in stationery shops). The hair is then statically charged by friction and its tapered point is used to catch the electron-transparent piece and deposit it onto the membrane of the MEMS chip as shown in the micrographs of Figure 1h,i. For slight repositioning of the sample upon the membrane, the hair tool can be washed in isopropanol. This serves to remove any residual static charge and allows manipulation of the sample without it attaching to the hair. The electron-transparent piece sticks firmly onto the MEMS e-chips and empirical experience (acquired by repeating this process several times) shows that smaller samples (on the order of the membrane dimensions of the MEMS chip, i.e., 50 µm × 50 µm) do not fall from the MEMS chip during sample loading into the electron-microscope, despite the holder turning upside-down during the loading steps. New practitioners of this procedure will likely find little difficulty when using the scalpel, though will need some small patience when using the hair to manipulate the sample position. A fair analogy would be threading a needle. With practice, the entire process from electropolished disk to positioned sample can take only about 15 min, which was indeed the case for the sample showcased in Figure 1.

Some final notes must be made regarding the sample preparation methodology above described. The final quality of the specimen will be dependent on the initial quality of the electropolished 3 mm disks as well as on the overall cutting procedure. While cutting will induce plastic deformation, and therefore dislocations at the cut edges, the ROI is actually the hole’s edge, which remains uncut. Although brittle thin samples can be easily cut with a scalpel, the AlMgZn(Cu) alloy used in this work is highly ductile [18] and cutting was also easily performed. Therefore, it is expected that the methodology works for electropolished metallic samples either brittle or ductile. For brittle materials, practitioners can expect that the scalpel, rather than cutting with plastic deformation through the sample, causes fractures and breaks beneath the blade, for the same end-result in ROI isolation. Ceramic materials were not tested in this work, but, given that 3 mm disks can be punched ultrasonically and subsequently mechanically thinned, dimpled and ion-polished (using a precise ion polishing system or PIPS) to electron-transparency, the proposed methodology may also be applicable for this class of materials.

### 3.2. Characterisation with S/TEM

**Figure 2:**
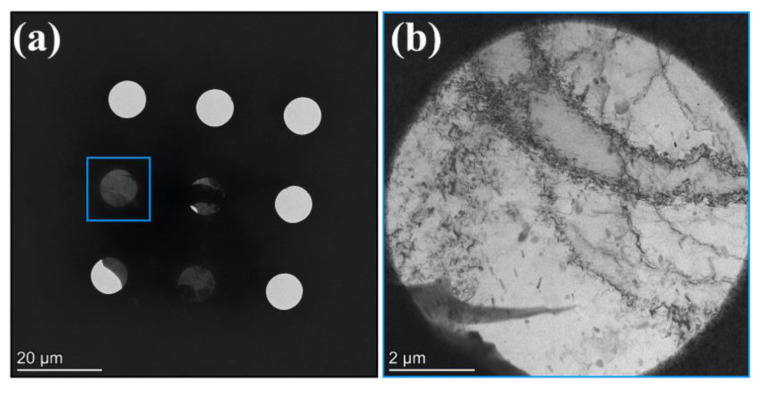
Bright-field TEM (BF-TEM) micrographs after the sample preparation procedure showing (**a**) an electron-transparent sample of the AlMgZn(Cu) alloy attached to the MEMS chip and (**b**) the sample lying over a hole (indicated by the blue square in (**a**)) on the MEMS chip.

The electron-transparent piece attached to the MEMS e-chip as shown in Figure 1i was loaded into the electron-microscope. Figure 2a shows a BF-TEM micrograph taken at low-magnification indicating that the sample was stationary on the MEMS chip during both loading procedures into the holder and into the electron-microscope. Figure 2a also indicates that the sample is covering four holes of the membrane of the MEMS e-chip. A high-magnification BF-TEM micrograph was taken from the hole indicated with a blue square in Figure 2a: as shown in Figure 2b, the sample is high-quality and electron-transparent, thus demonstrating the viability of the proposed sample preparation methodology.

It is worth emphasising that, when using the SEM-FIB for producing samples for MEMS e-chips, Pt is often deposited in specific areas of the sample in order to weld it onto the e-chip membrane [2]. This step may introduce a significant yield of Pt contamination onto the surfaces of the electron-transparent specimen, which may compromise the MEMS experiments. The same is expected to occur upon interaction of either Ga or Xe ion beams with the metallic specimens during the stages of trenching, milling, cutting and polishing within the SEM-FIB. Conversely, in the several steps of the sample preparation methodology reported in this work neither Pt, Ga nor Xe are used; therefore, the sample will be free of those contaminants when compared to samples made within the SEM-FIB.

### 3.3. Paint Bake of AlMgZn(Cu) Alloy within a S/TEM

**Figure 3:**
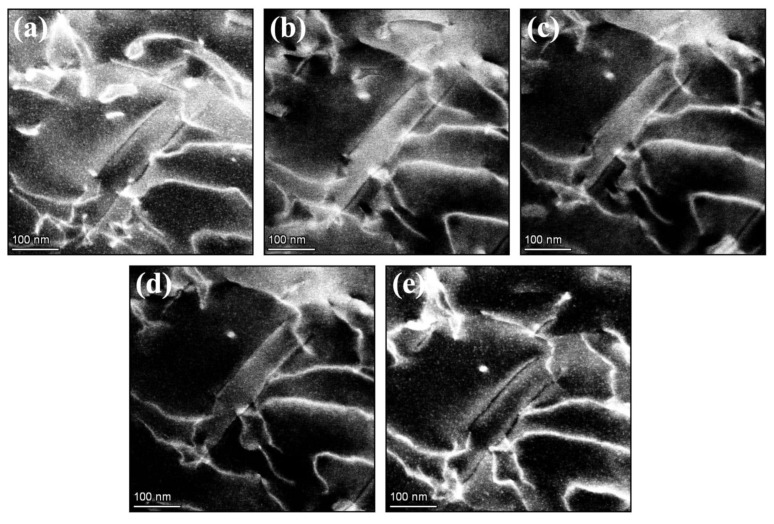
Heat treatment of the AlMgZn(Cu) alloy in situ within a scanning/transmission electron microscope (S/TEM) using the MEMS chip. The set of micrographs (**a**–**e**) and show the microstructural evolution of the AlMgZn(Cu) alloy as a function of time with the low-angle annular dark-field (LAADF) detectors. Micrograph (**a**) was taken prior to paint bake whilst micrographs (**b**), (**c**), (**d**) and (**e**) were taken at 200, 400, 800 and 1200 s, respectively. The corresponding bright-field STEM (BF-STEM) micrographs were reported in [18].

In order to evaluate both the quality and performance of the electron-transparent sample during a MEMS experiment a paint bake treatment was performed in situ in the STEM, which consisted of heating the AlMgZn(Cu) alloy up to 458 K for 1200 s. The LAADF micrograph in Figure 3a shows the microstructure of the alloy prior to paint bake, which is composed of nanometer-sized Guinier–Preston zones (or simply GP Zones) and a high density of dislocations, given that the alloy was 2% deformed. Note that this is similar microstructure to that found in a conventionally produced AlMgZn(Cu) alloy [18].

Upon paint bake, the microstructural evolution of the AlMgZn(Cu) alloy was monitored in real-time using the BF-STEM and LAADF detectors, the latter of which is shown in the micrographs in Figure 3b–e, the former being reported in [18]. A reorganization of the initial dislocation structure was observed to take place, including complete annihilation of pre-existing dislocations. During the experiment, the sample experienced minimal drift, only on the order of few nanometers, which was easily corrected manually with stage movement.

**Figure 4:**
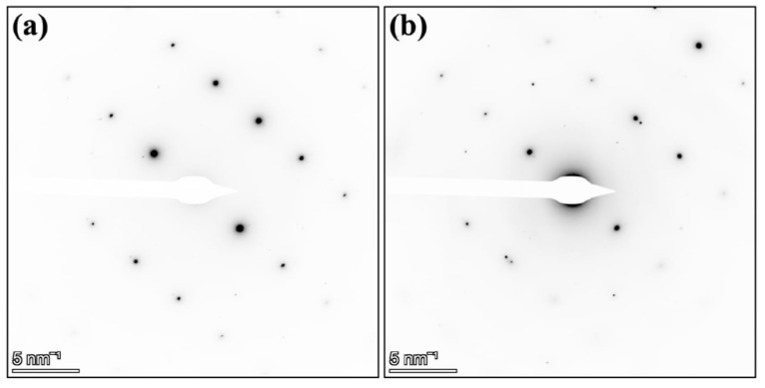
Selected-area electron diffraction (SAED) patterns of the AlMgZn(Cu) alloy oriented along [112] zone-axis (**a**) before and (**b**) after paint bake treatment. The additional spots in (**a**,**b**) are due to dispersoid phases not in the field of view. The field of view of the SAED patterns correspond to the selected-area aperture covering the whole grain in Figure 3a.

The SAED patterns shown in Figure 4a,b exhibit the microstructure of the AlMgZn(Cu) alloy before and after the paint bake treatment. It is worth emphasising that the SAED patterns before and after the experiment do not show any Debye–Scherrer rings commonly associated with polycrystalline nanometer-sized artefacts introduced by Pt and Ga contamination when using samples produced via the SEM-FIB technique. As this is an experiment performed with an electron-transparent lamella at the nanoscale, the results may not correspond directly to those reported by Stemper, where heat treatment was performed in bulk specimens [18].

### 3.4. Post-Experiment Impurity Analysis

**Figure 5:**
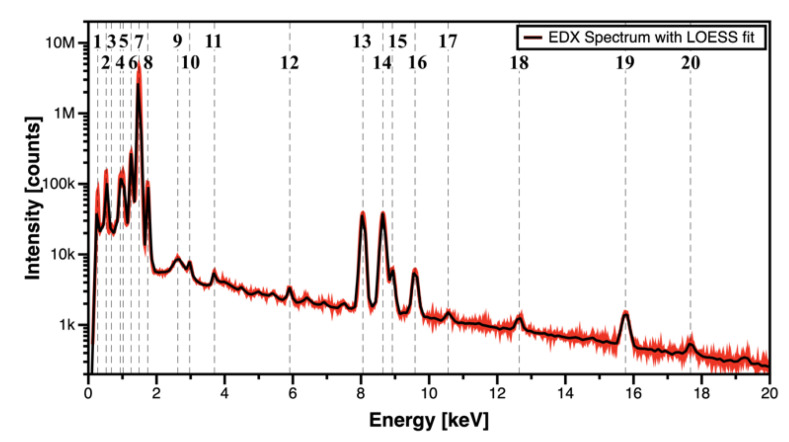
STEM energy dispersive X-ray (EDX) raw spectrum acquired from the whole area covered in Figure 4b. Note: the y-scale was set to logarithmic in order to maximize visualisation smaller peaks.

STEM-EDX was used to analyse the elements present in the sample after the in situ STEM paint bake treatment. Figure 5 shows a long-exposure (2 h) STEM-EDX raw spectrum collected from the whole area corresponding to the micrograph in Figure 4b. The intensity axis (y-axis) of the STEM-EDX plot in Figure 5 was set to logarithmic scale in order to better evaluate the presence of minor elemental peaks and a locally estimated scatterplot smoothing (LOESS) fit was used to better identify the peaks’ positions in the energy axis (x-axis). In order to define the relevance of a peak with respect to the background noise, the relative intensity of each identified peak was calculated against the strongest signal peak, e.g., the Al Kα peak index 7 located at 1.487 keV. Peaks with less than 0.01% of relative intensity were not considered.

Using this methodology, 20 peaks were identified in the STEM-EDX raw spectrum. The peaks’ indexes, as well as their energy and relative intensity, were extracted from the plot. The results are shown in Table 1 and the accuracy of the STEM-EDX detector is noted. Most of the peaks are confirmed to have an energy match (with their expected position using reference values in the software Velox) in the third decimal place. Given such accuracy, the absence of contaminants such as Pt (Lα = 9.442 keV and Mα = 2.050 keV) and Ga (Kα = 9.251 keV and Lα = 1.098 keV) is remarkably noted. Minor impurities were identified as coming from the alloy production: Ca and Zr with extremely low relative intensity: 0.13% and 0.01% respectively. The presence of O is expected as Al self-passivates. Due to overlap between multiple different elements, the peaks corresponding to labels 3, 9, 10, 12, 17 and 18 were not properly identified, although their relative intensity is below 1%; therefore, we assume these impurities are from the inner microscope electronics, holder, e-chip or background noise.

The only contaminant observed with relative intensity of 1.6% was the element C (peak label 1). It is well known that EDX precludes the identification of the element C [19], but the presence of this small peak can indeed be attributed to carbonaceous contamination either in the surface of the AlMgZn(Cu) sample or in the MEMS e-chip. However, this C contamination can be mitigated with the use of plasma cleaning, which was not applied in this work. Regardless of the minor C contamination and the presence of small impurities, the experiment and its outcomes were not in any way affected as confirmed by the detailed post-paint bake electron-microscopy analysis. No artefacts were observed to nucleate and grow on the alloy microstructure as a result of the experiment.

## 4. Conclusions

An alternative method for producing good-quality and implantation-free electron-transparent samples for MEMS experiments in situ within a S/TEM was introduced in this paper. The method consisted of using electropolished 3 mm disks from metallic samples with an electron-transparent hole in the center. The disk is then subjected to a set of precise cuts in order to separate the electron-transparent region into smaller pieces of around ≈50 µm. The electron-transparent piece can be transferred to pristine MEMS e-chips, with a high-quality animal hair used as a micrometer-sized manipulation tool.

The introduced methodology is faster than SEM-FIB and it allows the sample preparation of multiple samples from only one 3 mm electropolished disk. A paint bake experiment of an AlMgZn(Cu) alloy was performed in order to attest the quality of the sample and its stability during an in situ STEM experiment. Yield of minor impurities were observed to come from the Al alloy itself rather than the sample preparation method. The only minor extrinsic contamination observed was C, which is commonly identified when using the EDX technique for elemental estimation. None of the minor impurities nor C contamination were observed to affect the results of the paint bake experiment as no artefacts were observed to form and evolve in the sample.

This sample preparation methodology works well for both ductile and brittle metallic 3 mm electropolished disks, but it has not been yet tested for 3 mm dimpled and ion-polished ceramic discs.

## Figures and Tables

**Table 1:** STEM-EDX impurity analysis of the paint baked Al alloy.

Peak Index	Measured (keV)	Expected (keV)	Relative‡ (%)	Identified Element
1†	0.273	0.280	1.60	C Kα
2	0.521	0.524	3.20	O Kα
3†	0.675	-	0.61	Multi. Elements
4	0.933	0.929	2.89	Cu Lα
5	1.018	1.012	3.34	Zn Lα
6	1.253	1.254	6.34	Mg Kα
7	1.487	1.487	100	Al Kα
8	1.742	1.742	2.31	Si Kα
9†	2.623	-	0.21	Multi. Elements
10†	2.971	-	0.18	Multi. Elements
11†	3.699	3.691	0.13	Ca Kα
12†	5.910	-	0.08	Multi. Elements
13	8.055	8.048	0.09	Cu Kα
14	8.644	8.639	0.09	Zn Kα
15	8.921	8.907	0.14	Cu Kβ
16	9.587	9.574	0.14	Zn Kβ
17†	10.558	-	0.04	Multi. Elements
18†	12.643	-	0.03	Multi. Elements
19†	15.764	15.775	0.04	Zr Kα
20†	17.671	17.667	0.01	Zr Kβ

† Note 1: all the identified impurities have low relative intensity (i.e., the signal from impurities are comparable to noise). ‡ Note 2: the relative intensity was calculated with respect to the most intense peak in the spectrum (the Al Kα peak index 7).

## Data Availability

The raw/processed data required to reproduce these finding cannot be shared at this time as the data also forms part of an ongoing study.
